# Urokinase-type plasminogen activator receptor (uPAR) expression enhances invasion and metastasis in RAS mutated tumors

**DOI:** 10.1038/s41598-017-10062-1

**Published:** 2017-08-24

**Authors:** Concetta Di Mauro, Ada Pesapane, Luigi Formisano, Roberta Rosa, Valentina D’Amato, Paola Ciciola, Alberto Servetto, Roberta Marciano, Roberta Clara Orsini, Francesca Monteleone, Nicola Zambrano, Gabriella Fontanini, Adele Servadio, Giuseppe Pignataro, Lucia Grumetto, Antonio Lavecchia, Dario Bruzzese, Antonino Iaccarino, Giancarlo Troncone, Bianca Maria Veneziani, Nunzia Montuori, Sabino De Placido, Roberto Bianco

**Affiliations:** 1 Department of Clinical Medicine and Surgery, University of Naples “Federico II”, Naples, Italy; 2 Department of Translational Medical Sciences, University of Naples Federico II, Naples, Italy; 3 Department of Molecular Medicine and Medical Biotechnologies, University of Naples Federico II, Naples, Italy; 4 CEINGE Biotecnologie Avanzate S.C.aR.L, Naples, Italy; 5 Division of Pathology, Department of Surgery, University of Pisa, Pisa, Italy; 6 Department of Neuroscience, Reproductive Science and Dentistry, Division of Pharmacology, School of Medicine, “Federico II” University of Naples, Naples, Italy; 7 Department of Pharmacy, University of Naples Federico II, Naples, Italy; 8 Department of Public Health, Federico II University, Naples, Italy

## Abstract

The urokinase-type plasminogen activator receptor (uPAR) is a GPI-anchored cell membrane receptor that focuses urokinase (uPA) proteolytic activity on the cell surface. Its expression is increased in many human cancers, including non-small cell lung cancer (NSCLC) and colorectal cancer (CRC), and correlates with a poor prognosis and early invasion and metastasis. uPAR is able to control, through a cross-talk with tyrosine kinase receptors, the shift between tumor dormancy and proliferation, that usually precedes metastasis formation. Therefore, we investigated the role of uPAR expression in RAS mutated NSCLC and CRC cells. In this study we provided evidence, for the first time, that RAS mutational condition is functionally correlated to uPAR overexpression in NSCLC and CRC cancer cell lines and patient-derived tissue samples. Moreover, oncogenic features related to uPAR overexpression in RAS mutated NSCLC and CRC, such as adhesion, migration and metastatic process may be targeted, *in vitro* and *in vivo*, by new anti-uPAR small molecules, specific inhibitors of uPAR-vitronectin interaction. Therefore, anti-uPAR drugs could represent an effective pharmacological strategy for NSCLC and CRC patients carrying RAS mutations.

## Introduction

The urokinase-type plasminogen activator receptor (uPAR) is a GPI-anchored cell membrane receptor, composed by three homologous domains (DI, DII, DIII). Its main function is focusing of urokinase (uPA) proteolytic activity, responsible for degradation of extracellular matrix (ECM) components, on the cell surface^1^. uPAR expression is increased in many human cancers and correlates with a poor prognosis and early invasion and metastasis^2^. Elevated levels of uPAR have been also reported in primary tumors and serum of non-small cell lung (NSCLC)^3, 4^ and colorectal (CRC) cancer^5, 6^. uPAR can be shed from the cell surface by a specific phospholipase, and soluble uPAR (suPAR) has been detected in human plasma and urine^7^.

Besides uPAR’s traditional role in the regulation of proteolysis, its involvement in proteolysis-independent activities has been deeply demonstrated. Indeed, uPAR is an adhesion receptor, as it binds vitronectin (VN), an abundant component of provisional extracellular matrix (ECM)^8^. This direct interaction between uPAR and VN is critical for triggering changes in cell morphology, migration and signaling^9^ and seems to be an important requirement for the induction of epithelial mesenchymal transition (EMT)^10^. In addition, uPAR regulates cellular adhesion, migration, proliferation and survival^11, 12^, through interactions with other transmembrane receptors, such as integrins, G-protein-coupled chemotaxis receptors and tyrosine kinase receptors (RTKs)^13^.

uPAR, through integrins interaction, plays a crucial role in activating Src, FAK and paxillin, critical mediators of cell migration; particularly, paxillin acts as an adaptor protein, by binding integrins to FAK and Src kinases^14^. By interacting with chemotaxis receptors for fMet-Leu-Phe (fMLF-Rs), uPAR mediates both uPA- and fMLF-dependent cell migration^15^. Among RTKs, uPAR is implicated in an extensive cross-talk with epidermal growth factor receptor (EGFR) that mediates the shift from tumor cell dormancy to proliferation^16^. Indeed, through interaction with α_5_β_1_ integrin, uPAR can induce a ligand-independent EGFR signaling, that causes robust RAS/ERK activation; similarly, ERK can drive uPAR expression through activator protein 1 (AP-1) transcription factor^17^. RAS mutations, which disable the intrinsic GTPase activity promoting the oncogenic potential and increasing the activation of PI3K/AKT and MAPK pathways, represent a common mechanism of intrinsic resistance to EGFR inhibitors in NSCLC and CRC^18^. Therefore, NSCLC and CRC patients whose tumors carry mutations in RAS genes do not benefit from anti-EGFR drugs and nowadays are still orphans of molecular targeted anticancer therapy^19–21^. The identification of alternative pathway sustaining the RAS driven tumor progression represents a clinical challenge as well as an urgent need.

Therefore, we investigated the correlation between uPAR overexpression and RAS mutational status in NSCLC and CRC cell lines as well as in cancer tissue specimens. In addition, we examined the ability of newly-identified small molecules targeting uPAR^22^ to inhibit uPAR-mediated effects in RAS mutated NSCLC and CRC *in vitro* and *in vivo*, to obtain new promising treatments.

## Results

### uPAR expression is associated with RAS mutations in NSCLC and CRC patients

We evaluated by immunohistochemistry uPAR expression in NSCLC and CRC tumor samples characterized by different RAS mutational statuses. The NSCLC patient cohort included 102 RAS wild-type and 98 RAS mutated samples; the CRC patient cohort included 57 RAS wild-type and 51 RAS mutated samples. Patients features are depicted in Supplementary Table S1.

uPAR expression was significantly associated with RAS mutational status both in the NSCLC (p < 0.001) and in the CRC cohorts (p < 0.001): 49/98 (50%) RAS mutated NSCLC samples resulted positive for uPAR expression with 33/98 (33.7%) of samples showing strong uPAR expression (score > 3), while only 24/102 (24%) RAS wild-type NSCLC samples showed positivity for uPAR expression with 10/102 (9.8%) of samples achieving IHC score up to 3 (Table 1). Concerning CRC, uPAR expression was found in 33/51 (64.7%) RAS mutated samples with 20/51 (39.2%) strong positivity; in RAS wild-type samples only 15/57 (26.3%) showed positivity and 12/57 (21%) high positivity (score > 3) for uPAR expression (Table 1). In more details, in the NSCLC cohort, the presence of uPAR expression showed values of Sensitivity and Specificity equal, respectively, to 0.50 (95% Confidential Interval -C.I.- 0.40 to 0.60) and 0.76 (95% C.I. 0.69 to 0.84). Sensitivity and Specificity of uPAR expression in the CRC cohort were equal to 0.64 (95% C.I. 0.51 to 0.78) and 0.74 (95% C.I. 0.61 to 0.84).

**Table 1:** Association between RAS mutational status and uPAR expression.

	RAS status	uPAR expression			
		Negative	Positive	Score < 3	Score > 3
NSCLC	WT (102)	78 (76.5%)	24 (23.5%)	92 (90.2%)	10 (9.8%)
	MUT (98)	49 (50.0%)	49 (50.0%)	65 (66.3%)	33 (33.7%)
CRC	WT (57)	42 (73.7%)	15 (26.3%)	45 (78.9%)	12 (21.1%)
	MUT (51)	18 (35.3%)	33 (64.7%)	31 (60.8%)	20 (39.2%)

When considering strong uPAR expression (score > 3), Sensitivity and Specificity in the NSCLC cohort were equal to 0.34 (95% C.I. 0.25 to 0.43) and 0.90 (95% C.I. 0.84 to 0.96). In the CRC cohort, a score greater than 3 was associated to a Sensitivity of 0.39 (95% C.I. 0.25 to 0.53) and a Specificity of 0.79 (95% C.I. 0.68 to 0.89). Supplementary Figure S1 shows representative images of immunostaining for uPAR in NSCLC and CRC RAS mutated samples expressing strong positivity (score > 3) compared to RAS wild-type samples negative for uPAR expression.

### uPAR-dependent pathways are differentially modulated in RAS mutated cancer cells

In order to verify the existence of the same correlation between uPAR expression and RAS mutations in *in vitro* models, we first investigated uPAR expression in a panel of human NSCLC and CRC cell lines characterized by different RAS status. The cell lines features are depicted in supplementary Table S2.

**Figure 1:**
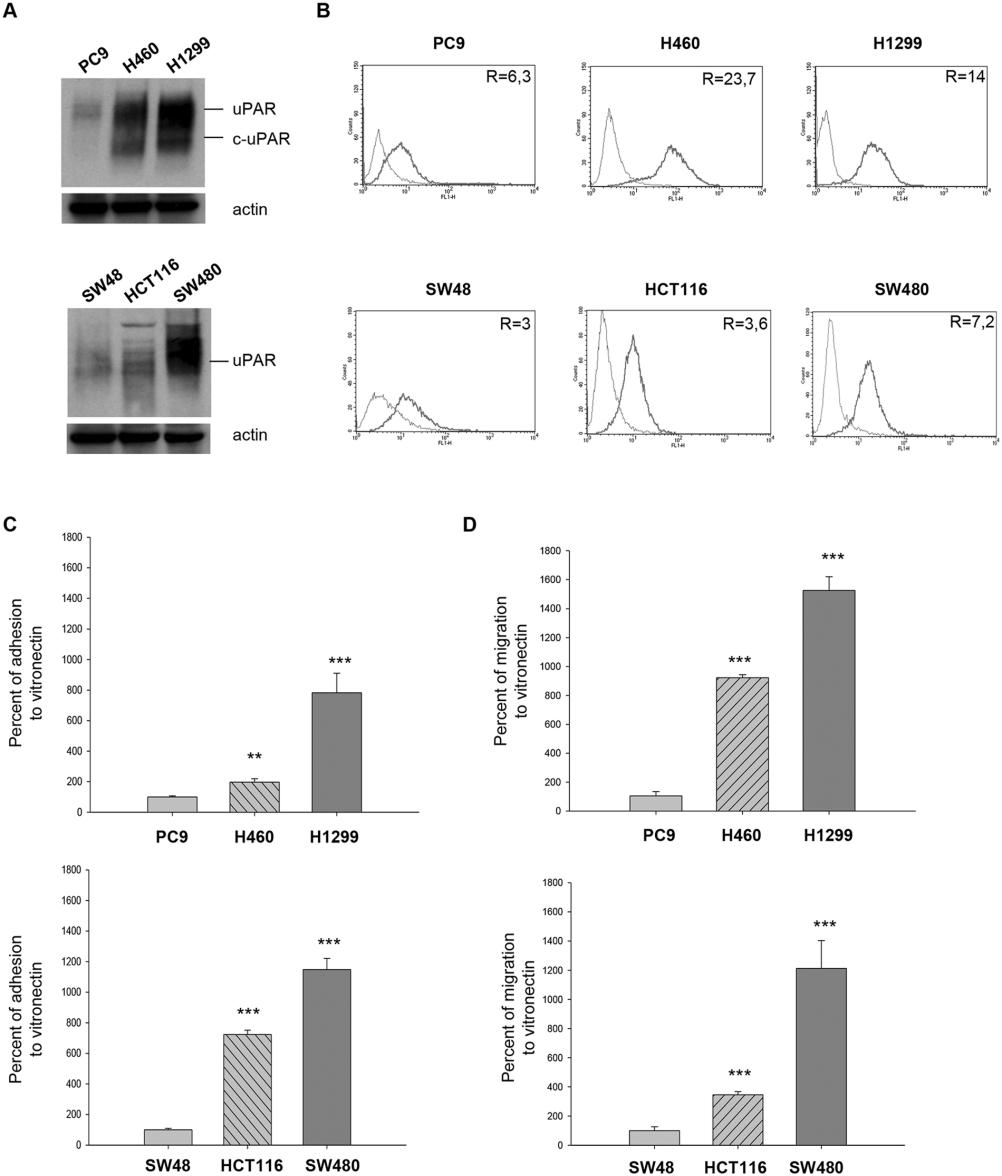
uPAR expression and functions in NSCLC and CRC cells, characterized by different RAS mutational status. **(A,B)** Western blot and cytofluorimetric analysis of uPAR expression in three NSCLC cell lines (PC9, H460, H1299) and in three CRC cell lines (SW48, HCT116, SW480). All immunoblot bands are cropped, full-length blot images are provided in Supplementary Figure S5. **(C,D)** Percent of adhesion and migration to VN in NSCLC and in CRC cell lines. Data represent the mean (±SD) of three independent experiments, each performed in triplicate. Asterisks indicate statistical significance of analyzed cellular processes in RAS mutated compared with RAS wild-type cell lines considered as 100%, determined by the Student t-test (**P < 0.005; ***P < 0.001).

Western blot analysis revealed that the expression of uPAR was higher in RAS mutated compared to RAS wild-type cell lines, both in NSCLC and CRC models (Supplementary Figure S2). In addition, NSCLC RAS mutated cell lines showed increased expression of cleaved uPAR (c-uPAR) (Supplementary Figure S2), the truncated form of uPAR able to interact with fMLF receptors and to induce chemotaxis^15^. For further studies, we selected one RAS wild-type and two uPAR overexpressing RAS mutated cell lines for each cancer model. In these selected cells, we confirmed uPAR expression by both Western blot (Fig. 1A) and cytofluorimetric analysis of surface receptors (Fig. 1B). The mean fluorescence intensity of cells incubated with anti-uPAR antibody or isotype control (non-immune IgG) and ratio values are reported in Supplementary Table S3.

We then evaluated the effect of uPAR overexpression on the main uPAR mediated cellular functions, such as adhesion and migration to VN^14^. The adhesion to VN was significantly higher in NSCLC RAS mutated cell lines such as H460 (p < 0.005) and H1299 (p < 0.001) than in RAS wild-type PC9 cells (Fig. 1C, top); also CRC RAS mutated HCT116 (p < 0.001) and SW480 (p < 0.001) cell lines showed higher adhesion to VN than RAS wild-type cell line SW48 (Fig. 1C, bottom). The RAS mutated and uPAR overexpressing cell lines showed a significant increase in migration to VN compared to RAS wild-type cell lines, both in NSCLC (Fig. 1D, top) and CRC (Fig. 1D, bottom) (p < 0.001).

**Figure 2:**
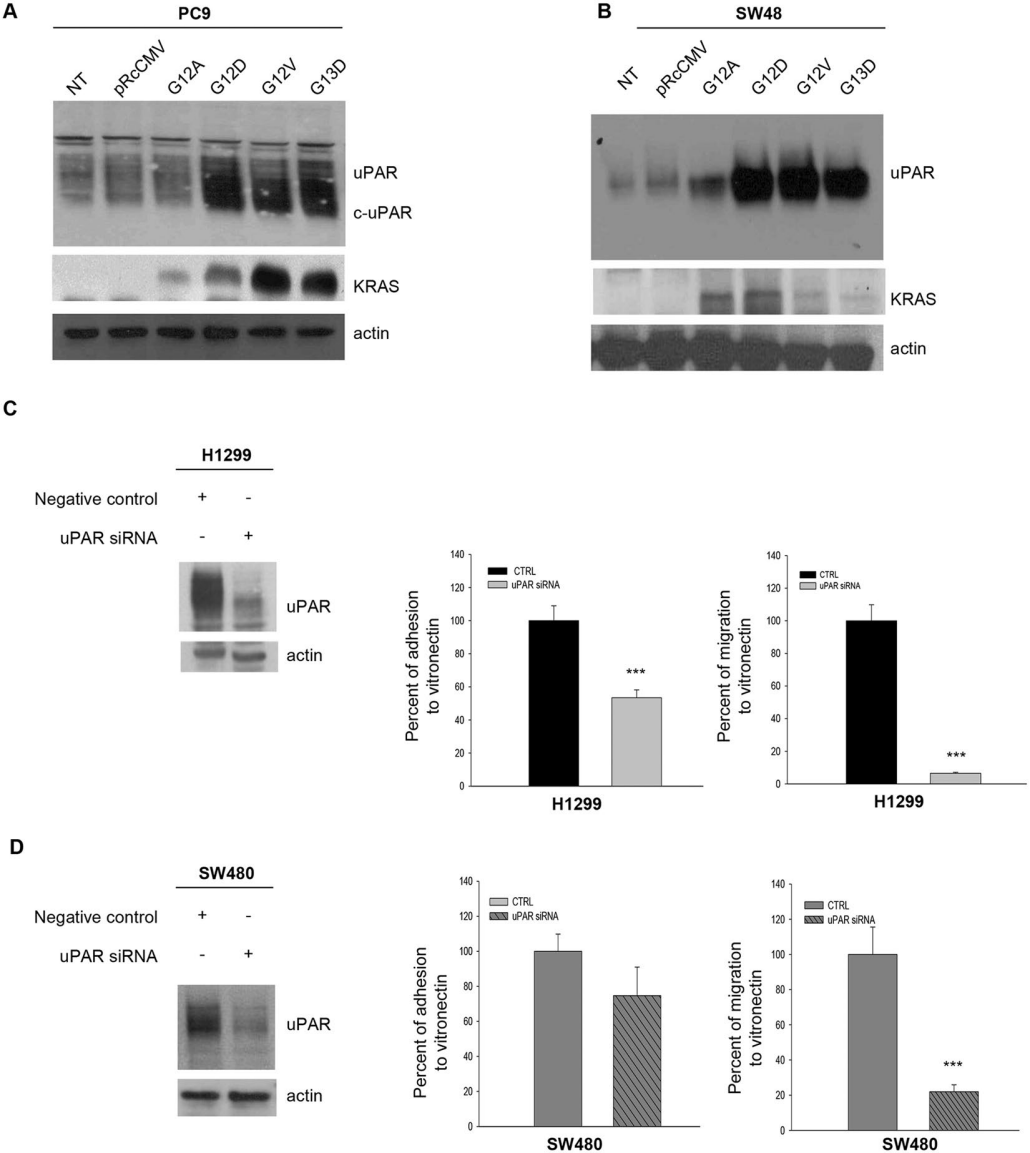
Effects of RAS mutated expression in RAS wild-type cells and uPAR-silencing in RAS mutated cells. **(A,B)** Western blot analysis of uPAR and RAS expression in RAS wild-type PC9 cells transiently transfected and in SW48 cells stably transfected with RAS mutated plasmids; we used not transfected (NT) or empty vector (pRcCMV) transfected parental cell lines as controls. **(C,D)** H1299 and SW480 cells were transfected with uPAR or control siRNA as described in Methods. 48hr after the transfection the cells were seeded in DMEM and the adhesion and migration to VN assay was perfomed. Each bar represents the percentage of adhesion or migration to VN ± SD of three independent experiments, each performed in triplicate. uPAR knockdown was confirmed by immunoblot analysis of cell lysates from plates 48hr after the transfection. All immunoblot bands are cropped, full-length blot images are provided in Supplementary Figure S5. Bars, SDs. Asterisks indicate statistical significance, as determined by the Student t-test (***P < 0.001).

In order to investigate how RAS activation could affect uPAR expression, we transfected four plasmids carrying different RAS mutations (G12A, G12D, G12V, G13D) in low uPAR expressing PC9 cell line. As reported by Varmus *et al*.^23^, the concomitant stable expression of RAS and EGFR mutations leads to increased cell death in PC9; therefore we evaluated uPAR expression only after a transient transfection. As shown in Fig. 2A, the RAS mutated gene transfection was coupled with a strong induction of uPAR and c-uPAR expression in PC9 cell line, suggesting a direct link between RAS mutational status and uPAR overexpression. No significant changes were observed in control cells or transfected with the corresponding empty vector pRcCMV. uPAR overexpression was obtained also in SW48 cells stably transfected with the same RAS mutated plasmids (Fig. 2B).

NSCLC and CRC RAS mutated cell lines showed increased adhesion and migration to VN; to analyze whether the increased cellular functions of RAS mutated cell lines are partially due to uPAR overexpression, we performed uPAR silencing in H1299 and SW480 cells. As shown in Fig. 2C and D, a strong reduction of uPAR expression was obtained in RAS mutated cells transfected with a uPAR specific siRNA, as revealed by Western blot analysis. The uPAR silenced H1299 cells showed significant reduction of adhesion and migration to VN (p < 0.001); uPAR silencing in SW480 cells induced a significant reduction of migration (p < 0.001) and a slight reduction of adhesion to VN (Fig. 2D). As shown in Supplementary Figure S3, the uPAR silenced H1299 cells displayed also reduction of FAK, Src and paxillin phosphorylation.

### uPAR inhibition impairs adhesion, migration and cell signaling in RAS mutated cancer cells

We investigated the ability of new small molecules targeting uPAR-VN binding site, C6 and C37, previously identified and validated by Rea and colleagues, to inhibit uPAR-mediated functions in RAS mutated NSCLC and CRC cell lines. As already published, both compounds targeted two residues of uPAR chemotactic domain (aa. 88–92), critical for uPAR binding to VN and uPAR interaction with the f-MLFRs^22^.

**Figure 3:**
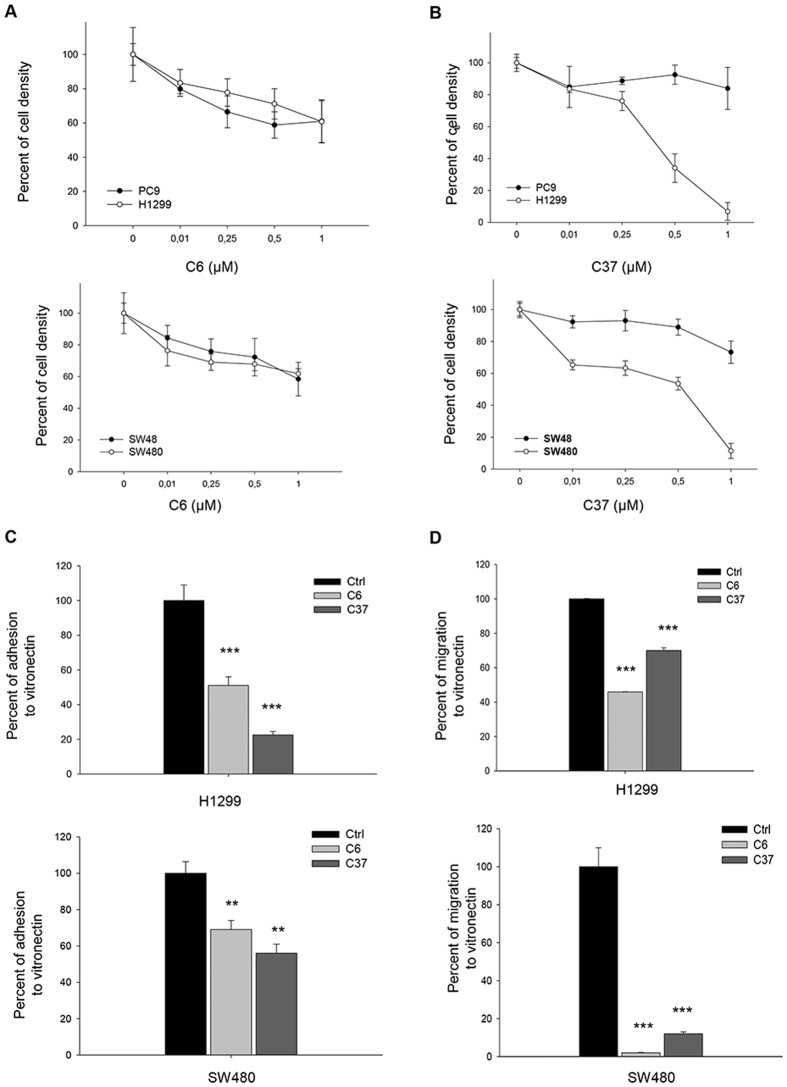
Effects of two uPAR inhibitors C6 and C37 on uPAR-mediated cellular functions. **(A,B)** Percent of cell density in NSCLC (top panel) and CRC (bottom panel)cell lines treated with increasing concentration of C6 (left) and C37 (right). **(C,D)** Percent of cellular adhesion and migration to VN in RAS mutated H1299 (top panel) and SW480 (bottom panel) cell lines treated with fixed doses of C6 and C37 (2,5 μM). Data represent the mean ( ± S.D.) of three independent experiments, each performed in triplicate, compared with control (cells treated with DMSO). Error bars indicate S.D. (**P < 0.005; ***P < 0.001).

Cell density assays were performed in RAS-mutated H1299 and SW480 and RAS wild-type PC9 and SW48 cell lines treated with increasing concentrations of C6 and C37. C6 treatment had no differential effects on RAS mutated compared to RAS wild-type cell lines both in NSCLC (Fig. 3A, top panel) and in CRC (Fig. 3A, bottom panel). C37 treatment reduced cell density in a concentration-dependent manner, with IC_50_ ranging between 0.25 and 0.5 μM for H1299 and between 0.5 and 1 μM for SW480; the IC_50_ was not achieved in RAS wild-type PC9 (Fig. 3B, top panel) and SW48 (Fig. 3B, bottom panel) cell lines In PC9 cells, that shows EGFR addiction because of the A746-A750del in exon 19^24^, we also tested the combination of C6 or C37 with the EGFR tyrosine kinase inhibitor (TKI) gefitinib in cell density assays. Neither C6 nor C37 did modify the dose-response curve to gefitinib in a statistically significant manner (Supplementary Figure S4; linear regression analysis: C6, P = 0.29; C37, P = 0.92).

**Figure 4:**
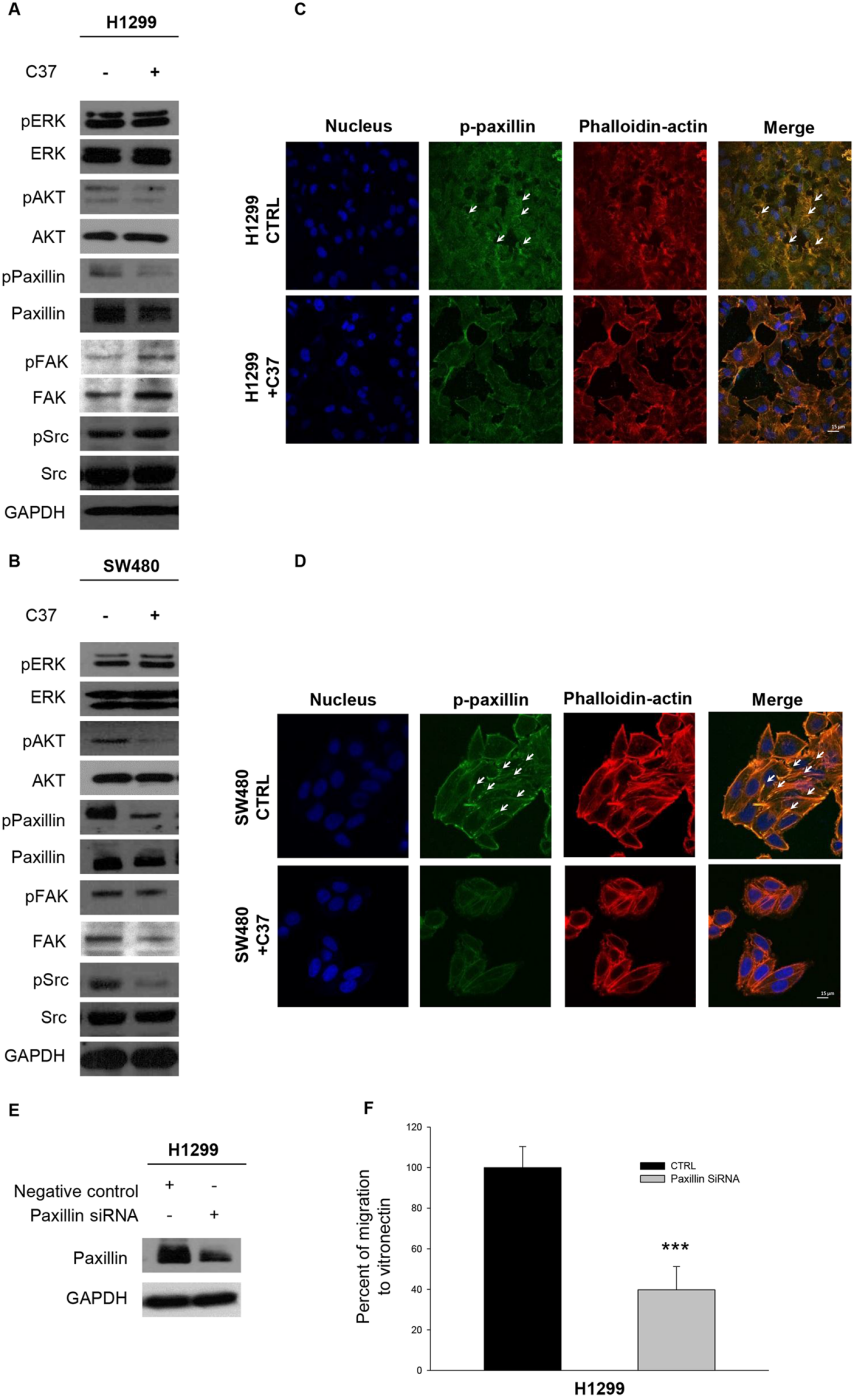
C37 treatment modulates p-Paxillin in H1299 and SW480 cell lines. **(A,B)** Immunoblot analysis on total cell lysates from H1299 and SW480 treated with 2,5 μM C37 for 24 hours. All immunoblot bands are cropped, full-length blot images are provided in Supplementary Figure S5. **(C,D)** H1299 and SW480 cells were plated and treated with 2.5 μM C37 for 24 hours. Monolayers were subjected to immunofluorescent staining of p-paxillin (green), actin (red) and nuclei (blue) and confocal microscopy analysis as described in Methods. Merged column images show overlapping signal. The white arrows indicate cytoskeleton organization. Scale bars, 15 mm. (**E**) Western blot analysis of H1299 cells 48 h after transfection with paxillin or control siRNA as described in Methods. (**F**) Percent of cellular migration to VN in H1299cells 48 h after transfection with paxillin or control siRNA as described in Methods.

Moreover, we evaluated adhesion to VN in H1299 and SW480 lines in response to fixed dose of C6 and C37. As shown in Fig. 3C, both compounds induced reduction of adhesion to VN, as compared to control cells, in H1299 (Fig. 3C, top panel) as well as in SW480 (Fig. 3C, bottom panel); in particular C37 showed a stronger effect than C6 in inhibiting uPAR-mediated adhesion to VN in both cell lines. Migration assays were also performed in H1299 and SW480 cells treated with a fixed dose of C6 and C37; although both C6 and C37 treatment discouraged cell migration, C6 was more active than C37 for both H1299 (Fig. 3D, top panel) and SW480 (Fig. 3D, bottom panel) cell lines (p < 0.005). We evaluated the effects of C37 treatment on cell signaling in H1299 and SW480. Although Western blot analysis showed no significant modulation on activation of proliferation markers such as ERK and AKT, a reduction of paxillin activation, marker of focal adhesion^25^, was found (Fig. 4A and B). In order to characterize cytoskeleton dynamics in response to C37, we performed an immunofluorescence staining for phospho-paxillin and for F-actin in H1299 and SW480 cells. C37 treatment reduced paxillin phosphorylation and partially disorganized the cytoskeleton (Fig. 4C and D).

To better investigate the role of paxillin in uPAR-mediated migration to VN, we performed migration assays on H1299 cells after paxillin silencing. Western blot analysis demonstrated the efficacy of paxillin siRNA (Fig. 4E). As depicted in Fig. 4F, paxillin siRNA significantly reduced migration to VN of H1299 cells (p < 0.001).

### C37, an anti-uPAR small molecule, inhibits ***in vivo*** metastases formation

Our data suggest that uPAR overexpression in RAS mutated NSCLC and CRC cell lines is coupled with increased cellular functions such as adhesion and migration to VN. In order to analyze the overall effect of these findings, an *in vivo* experiment was performed in Balb/C nude mice xenografted with RAS mutated HCT116 cells. C37 doses used *in vivo* have been chosen taking into account the effective doses reported for the *in vitro* studies and applying the classical *In Vitro In Vivo* Correlation (IVIVC) analysis. In particular, following the Biopharmaceuitics Classification System, C37 can be included in class II, low solubility and high permeability, therefore a good IVIVC correlation is expected, unless dose is very high^26^. At the doses used in the present work the compound was detectable until 6 hours after administration.

**Figure 5:**
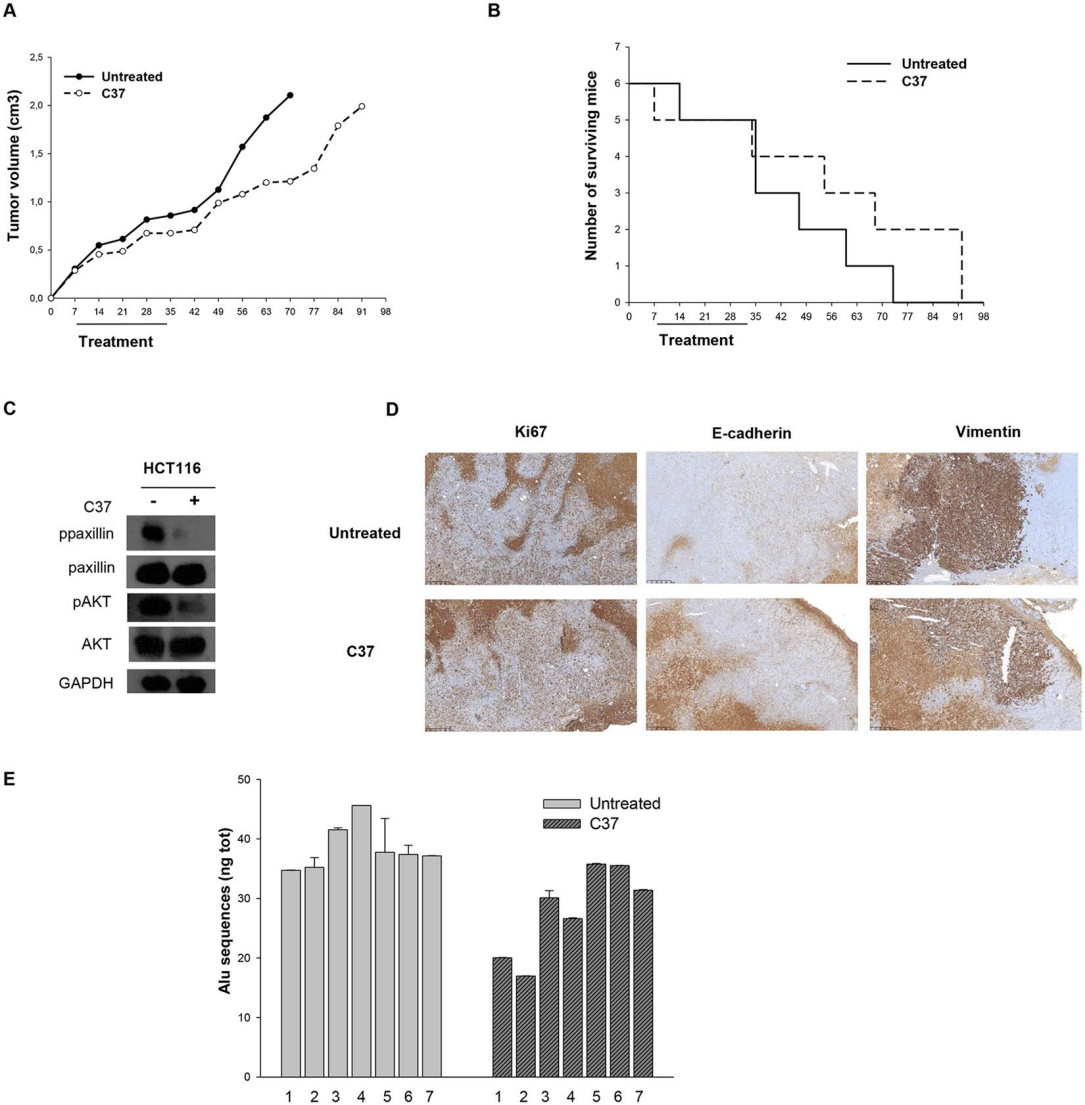
Effects of C37 on CRC tumor xenografts growth and lung metastases in mice. **(A)** Tumor volume of HCT116 xenografts in nude mice, randomized to receive C37, as described in the Methods section. **(B)** Number of surviving mice xenografted with HCT116 cells, after treatments with C37, as described in the Methods section. Median survival was 61.5 days in treated mice versus 41 days in control mice. **(C)** Western blot analysis on total lysates from HCT116 tumor specimens of mice sacrificed on day 21, after two weeks of treatment with C37. All immunoblot bands are cropped, full-length blot images for pPaxillin, Paxillin, pAKT and AKT are provided in Supplementary Figure S5. (**D**) Immunoistochemical analysis of Ki67, E-cadherin and vimentin expression on HCT116 tumor xenografts after treatments with C37 **(E)** Alu sequences quantification after artificial metastasis assay of HCT116 RAS mutated cells injected in tail vein of nude mice and treated with C37 or vehicle.

Untreated mice reached the maximum allowed tumor size, ca. 2 cm^3^, on day 70; at this time point, C37 treatment produced 39.5% of growth inhibition, even though it was not statistically significant (Fig. 5A). As shown in Fig. 5B, mice treated with C37 showed a slightly prolonged median survival compared with control mice with median survival in C37 treated mice of 61.50 vs 41.00 days in control mice (p = 0.29). We did not observe significant secondary effects such as diarrhea, weight loss, rash and behavior disorder in the mice treated with C37 compared to untreated mice (Supplementary Table S4). As already observed in the *in vitro* experiment, C37 reduced paxillin phosphorylation in total extracts from mice tumors. Although results of *in vitro* experiments showed no significant modulation of proliferation markers, C37 treatment resulted in reduction of AKT phosphorylation *in vivo* (Fig. 5C). Immunohistochemical analysis of mice xenografts did not show any difference in Ki67 staining (Fig. 5D); a switch from mesenchymal to epithelial phenotype was demonstrated by both increase of E-cadherin and reduction of vimentin expression after C37 treatment (Fig. 5D and Supplementary Table S5).

Since uPAR has been involved in metastatic process^2^, we investigated the effect of C37 in an artificial model of metastasis. HCT116 cells were injected in the tail vein of nude mice and treatments with C37 or vehicle (N = 7 per group) started immediately after cell injection, following the same schedule adopted for the tumor volume and survival experiments. At the end of the pharmacological treatments mice were sacrificed and DNA was extracted from lungs. Because of the high content of Alu sequences in human genomic DNA^27^, quantification of human cancer cells DNA in mice lungs was carried out through Alu sequences amplification by qRT-PCR. Human DNA was easily detected in all control mice whereas a statistically significant reduction of human DNA was observed in lungs of mice treated with C37 compared to control (p = 0.044), highlighting the efficacy of C37 treatment on *in vivo* metastatic process (Fig. 5E).

## Discussion

Patients affected by lung and colorectal cancer, carrying activating mutations in RAS, are characterized by poor prognosis and restricted therapeutic chances^28, 29^. Consequently, identifying alternative pathways sustaining the RAS mutated tumor phenotype represents an urgent need for clinicians.

Literature data indicated that uPAR is overexpressed in NSCLC and CRC tumors^3–6^ and it is involved in an extensive cross-talk RTKs, responsible for the induction of proliferation; moreover, the interaction of uPAR with vitronectin is able to drive the transmigration of cancer cells from blood circulation to tissues^12^. Therefore, we investigated whether uPAR could be a novel therapeutic target in RAS mutated NSCLC and CRC.

In our work, we observed that about 50% of RAS mutated NSCLC and 64.7% of RAS mutated CRC patients expressed uPAR protein at high or moderate levels. Interestingly, a statistically significant association between uPAR expression and RAS mutated status was found in both NSCLC and CRC patients. Furthermore, we provided evidence for a direct correlation between RAS mutational status and uPAR overexpression also in NSCLC and CRC cell lines. RAS mutated NSCLC and CRC cell lines express increased levels of uPAR, both in the full-length form, able to interact with EGFR and integrins^14^, and in the cleaved form, able to activate chemotaxis receptors^15^. uPAR overexpression in RAS mutated NSCLC and CRC cell lines corresponds to an increased adhesion and migration to VN, major component of provisional ECM associated to inflammation and tumor^13^. Conversely, uPAR silencing in RAS mutated NSCLC and CRC cells reduced adhesion and migration to VN, probably through inhibition of FAK, Src and paxillin. Beside its role in inducing ECM degradation through proteases activation, uPAR promotes metastasis formation mostly by engaging VN through Rac-1 activation^30, 31^ and by fostering EMT^9, 10^.

Thus, we assessed uPAR pharmacological inhibition by two specific inhibitors of uPAR-VN binding site as an alternative antitumor strategy in RAS mutated NSCLC and CRC. These uPAR inhibitors, named C6 and C37, small molecules identified by structure-based virtual screening, are able to disrupt uPAR binding to VN, thus blocking tumor cell growth, migration and invasion. The sensitivity to the compounds of the tested cell lines showed some difference that could be explained by their different localization in uPAR-VN binding site; indeed compound C6 mimics VN itself, extending into the uPAR-VN binding site, while C37 entirely fills the VN recognition pocket of the receptor^22^.

*In vitro* experiments showed a significant reduction of cell proliferation in RAS mutated cells after C37 treatment, as compared to RAS wild-type cells, while C6 did not exert any effect. Moreover it was observed a significant decrease of uPAR-mediated adhesion and migration to VN in RAS mutated cells treated with both compounds. Among uPAR interactors involved in adhesion and migration process, while uPAR silencing reduced phosphorylation of FAK, Src, and paxillin, as previously reported^32^, C37 treatment induced only a reduction of paxillin activation, influencing its interaction with microtubules at focal adhesions and perturbing microtubule dynamics. These data may suggest that paxillin is the main transducer involved in uPAR-mediated migration to VN, which is interfered by C37.

To evaluate the translational relevance of these findings, the effect of C37 was tested in RAS mutated CRC xenografted mice. Although C37 did not provide a statistical significant inhibition on tumor cells proliferation, it was able to significantly interfere with the metastasis process *in vivo*, discouraging the transmigration of cancer cells from blood circulation to the lungs.

Our results strengthen Rea and colleagues data^22^, and demonstrate, for the first time, that a small molecule against uPAR-VN binding site possesses anti-tumor activity in RAS mutated *in vivo* models. Target agents against uPA/uPAR interaction, proposed and tested in preclinical studies, were never advanced into the clinic, as they exerted a modest antitumor activity^33, 34^. Only an uPA inhibitor (Mesupron) advanced to clinical trials, with a slight improved progression free survival^35^, suggesting that the uPAR proteolytic function inhibition is not a viable strategy. Our data suggest for the first time an important role for uPAR in RAS mutated cancers and demonstrate that inhibiting the interaction between uPAR and VN could represent a useful strategy to counteract metastasis formation. Although C37 compound requires a chemical refinement in order to obtain a more active molecule, a clinical development of anti-uPAR drugs as a novel pharmacological therapy in human cancer carrying RAS activating mutations is warranted.

## Methods

### Immunohistochemical analysis

For immunohistochemical analysis, samples of NSCLC and CRC were collected from 2013 to 2015 in the Department of Surgical, Medical and Molecular Pathology and Critical Area, University of Pisa, Italy. Informed consent was obtained from all patients. Paraffin slides were de-paraffinized in xylene, rehydrated through graded alcohols and processed for immunohistochemistry (IHC) with uPAR antibody (Rabbit Polyclonal anti-uPAR antibody, Novus Biologicals, CO, USA). An observer evaluated the intensity, extend, subcellular distribution and number of positive cells.

There are not standardized criteria for uPAR staining evaluation. The staining intensity was graded as follows: 0, negative; + , weak;++, moderate; and +++ , intense; the proportion was graded according to the percentage of positive cells as follows: 0, negative; 1, 1–20%; 2, 21–50%; 3, 51–70%; 4, 71–100%. The intensity score and proportion score were summed in order to generate a total score. Total Score 0 defines negative expression, score ranging between 1 and 3 defines moderate expression and score greater than 3 high expression.

### Compounds and cell cultures

uPAR inhibitors C6 and C37^22^ were obtained from the NCI/DTP Open Chemical Repository, were dissolved in dimethyl sulfoxide (DMSO) and stored at −20 °C, at a concentration of 0.01 mol/L. For this study, a panel of immortalized NSCLC (PC9, HCC827, GLC82, H460, H1299, A549) and CRC (SW48, LS174T, HCT116, GEO, SW480, LoVo) cancer cell lines, obtained from the American Type Culture Collection, were used. Some experiments were performed on SW48 cells stably transfected with different pRcCMV plasmids containing KAS wild-type or mutant coding sequences. For a detailed description, see “RAS gene mutagenesis and RAS mutant transfection” methods and supplementary methods.

### RAS gene mutagenesis and RAS mutant transfection

The wild-type and mutant RAS expression vectors were generated by site-directed mutagenesis with the QuikChange™ II XL kit (Agilent, CA, USA). For a detailed description, see supplementary methods. RAS wild-type PC9 and SW48 cells were transfected with RAS mutant vectors; stable SW48 cell lines were established by selection of positive transfected clones grown in media containing G418.

### Western blot

Total protein extracts obtained from cell cultures or tumor specimens were resolved by 8–15% SDS-PAGE and probed with anti-human uPAR (kindly provided by Dr. G. Hoyer-Hansen, Finsen Laboratory, Copenhagen, Denmark), pAkt/Akt, pERK/ERK, p-paxillin/paxillin, pSrc/Src; pFAK/FAK; GAPDH(Cell Signaling Technologies, Beverly, MA, USA). Filters were further incubated for 30 minutes at room temperature with horseradish peroxidase (HRP)-conjugated anti-mouse or anti-rabbit antibodies (Bio-Rad, Milan, Italy). The reaction was detected by ECL, according to the manufacturer’s instructions.

### Flow cytometric analysis of surface molecules

NSCLC and CRC cell lines were harvested and incubated with 10 μg/ml of anti-uPAR (American Diagnostica, Greenwich, CT, USA) or isotype control antibodies for 1 h at 4 °C. This step was followed by a second incubation with a fluorescein isothiocyanate-labeled goat anti-rabbit IgG (Jackson ImmunoResearch, West Grove, PA, USA) for 30 min at 4 °C. Finally, the cells were washed and analyzed by flow cytometry using a FACScan (Becton–Dickinson, Mountain View, CA, USA). A total of 10^4^ events for each sample were acquired in all cytofluorimetric analyses.

### Cell Adhesion assay

Adhesion assays were conducted on 96-well flat bottomed plates for cell (Nunc, Roskild, Denmark) coated with VN (BD Biosciences, Franklin Lakes, NJ, USA) or with bovine serum albumin (BSA) in PBS as a negative control. NSCLC and CRC cells were plated into the wells and attached cells were fixed with paraformaldehyde in PBS and stained with crystal violet. Stain was eluted and the absorbance at 540 nm was measured by a spectrophotometer. In a separate set of experiments the cells were pre-incubated for 30 minutes at room temperature with uPAR inhibitors C6 and C37 or with DMSO, as a vehicle control. The results were expressed as percent of adhesion to VN with the amount of cell adhesion observed in PC9 or SW48 established as 100%.

### Migration assay

Cell migration assays were performed using Transwell polycarbonate filters BD Biosciences). The bottom of wells in a 24-multiwell plate were coated with VN or BSA in PBS, as a negative control; then NSCLC and CRC cells were seeded on filters in serum-free media. After 48 hours the cells on the underside of the transwell were fixed and stained with crystal violet. Absorbance at 540 nm was measured by a spectrophotometer with the amount of cell migration observed in PC9 or SW48 established as 100%.

In a separate set of experiment cells were seeded on filters and treated with uPAR inhibitors C6 or C37 or DMSO as a negative control. The amount of cell migration observed in negative control were established as 100%. Upper cell layer was removed with a cotton swab and cells on the underside of the transwell were fixed and stained with crystal violet. Absorbance at 540 nm was measured by a spectrophotometer with the amount of cell migration observed in PC9 or SW48 established as 100%.

In a separate set of experiment cells were seeded on filters in serum-free media and treated with uPAR inhibitors C6 or C37 or DMSO as a negative control. The amount of cell migration observed in negative control was established as 100%.

In a separate set of experiment cells were seeded on filters in serum-free media and silenced with paxillin siRNA or scramble as a negative control. The amount of cell migration observed in negative control was established as 100%.

### RNA interference

To knock-down uPAR orpaxillin, 2 × 10^5^ H1299 or SW480 cells were seeded in six-well plates and transfected with 50 nM uPAR- or paxillin-targeting or control siRNAs in antibiotic-free medium using Oligofectamine (Invitrogen), according to the manufacturer’s instructions. Cells were incubated for 48 h at 37 °C, 5% CO2, and then washed and lysed in 1% TX-100 or plated for adhesion and migration assays.

### Cell density assay

Cells (2 × 10^4^ cells per well) were grown in 24-well plates and exposed to increasing doses of C6 or C37. The percentage of cell density was determined using the 3-(4,5-dimethylthiazol-2-yl)-2,5-diphenyltetrazolium bromide (MTT) assay according to the manufacturer’s instructions. The dose response curves for each agent were determined at a fixed ratio based on the drug concentration causing 50% inhibition of cell proliferation.

### Immunofluorescence

For immunofluorescence assay on cancer cells, H1299 and SW480 cells (3 × 10^4^/well) were plated in 24-well plates, and treated with C37 (2.5 µM). After 24 hours of treatment, cells were stained for p-paxillin (Cell Signaling Technologies); Phalloidin-TRITC was used to visualize the actin cytoskeleton organization. Nuclei are stained in blue with Hoechst 33342.

### Nude mice colon cancer xenograft models

Five-week-old Balb/cAnNCrlBR athymic (nu+/nu+) mice (Charles River Laboratories, Milan, Italy) maintained in accordance with institutional guidelines of the University of Naples Animal Care Committee and in accordance with the Declaration of Helsinki were injected subcutaneously with HCT116 cells (10^7^ cells per mice) resuspended in 200 μl of Matrigel (BD Biosciences, Billerica, MA). Seven days after the tumor cell injection, tumor-bearing mice were randomly assigned (6 per group) to receive C37 treatment (200 μg Kg-1; i.p.) daily for four weeks. Tumor volume (cm^3^) was measured using the formula p/6larger diameter (smaller diameter)^2^, as previously reported^36^.

### Experimental metastasis assay

7 mice per group were inoculated with 3 × 10^5^ cells via tail vein injection, followed by C37 treatment (200 μg Kg-1; i.p.) for 21 consecutive days. At the end of the treatment, mice were sacrificed, lungs collected and DNA extracted using Illustra DNA Extraction Kit HT (GE Healthcare, Little Chalfont, UK). Human DNA in mouse lungs was measured by quantifying Alu sequences through PCR, as previously described^37^.

### Animal rights statement

All the experimental protocols were approved by the “University of Naples Animal Care Committee” (ethical approval protocol number 83). All applicable international, national, and institutional guidelines of the care and use of animals were followed.

### Human rights statement

Tumor samples from patients were managed at the Division of Pathology, Department of Surgery, University of Pisa, Italy. All experimental protocols were approved by “Ethical Committee Area Vasto Nord Ovest- University of Pisa”.

All procedures performed in studies involving human participants were in accordance with the ethical standards of the institutional and national research committee; informed consent in accordance with the Declaration of Helsinki was obtained from all patients or their legal guardians.

For further details concerning the Material and Methods used in this work, please see the Supplementary Methods section.

## Electronic supplementary material

Supplementary data
