# Supplementary material for: Urokinase-type plasminogen activator receptor (uPAR) expression enhances invasion and metastasis in RAS mutated tumors
Source: Sci Rep. 2017 Aug 24;7:9388. doi: 10.1038/s41598-017-10062-1 (PMC5571185; doi:10.1038/s41598-017-10062-1)
Supplement: Supplementary file 1 — Supplementary data [file 41598_2017_10062_MOESM1_ESM.pdf]

Urokinase-type plasminogen activator receptor (uPAR) expression enhances invasion and metastasis in RAS mutated tumors.

Concetta Di Mauro[1#], Ada Pesapane[2#], Luigi Formisano[1], Roberta Rosa[1], Valentina D'Amato[1], Paola Ciciola[1], Alberto Servetto[1], Roberta Marciano[1], Roberta Clara Orsini[1], Francesca Monteleone[3,4], Nicola Zambrano[3,4], Gabriella Fontanini[5], Adele Servadio[5], Giuseppe Pignataro[6], Lucia Grumetto[7], Antonio Lavecchia[7], Dario Bruzzese[8], Antonino Iaccarino[8], Giancarlo Troncone[8], Bianca Maria Veneziani[3], Nunzia Montuori[2], Sabino De Placido[1] , Roberto Bianco[1*].

[1]Department of Clinical Medicine and Surgery, University of Naples "Federico II", Naples, Italy; [2]Department of Translational Medical Sciences, University of Naples Federico II, Naples, Italy; [3]Department of Molecular Medicine and Medical Biotechnologies, University of Naples Federico II; [4]CEINGE Biotecnologie Avanzate S.C.aR.L., Naples, Italy; [5]Division of Pathology, Department of Surgery, University of Pisa; [6]Department of Neuroscience, Reproductive Science and Dentistry, Division of Pharmacology, School of Medicine, "Federico II" University of Naples, Naples, Italy; [7]Department of Pharmacy, University of Naples Federico II, Naples, Italy; [8]Department of Public Health, Federico II University, Naples, Italy.

[#] These authors contributed equally to this work.

***Correspondence to**: Roberto Bianco, M.D., Ph.D., Laboratory of Molecular Cancer Therapy, Department of Clinical Medicine and Surgery, University of Naples "Federico II", Via S. Pansini 5, 80131 Napoli, Italy. Tel: +39-081-7462061, Fax: +39-081-7462061; e-mail: robianco@unina.it

Running Title: uPAR overexpression in RAS mutated tumors.

Keywords: Urokinase-type plasminogen activator receptor, anti-EGFR drugs resistance, RAS, Non-small cell lung cancer, colorectal cancer.

Supplementary Figure S1

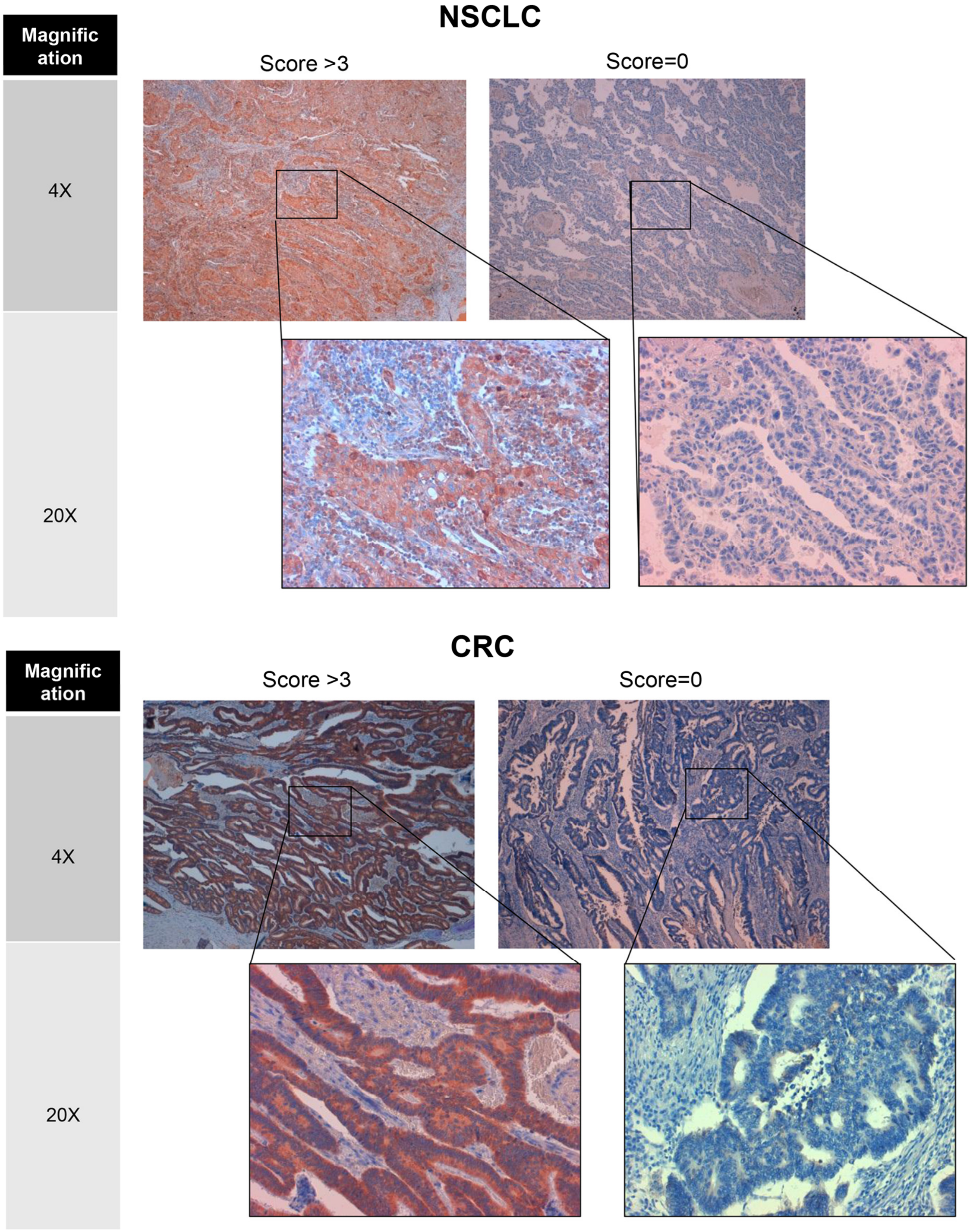

Supplementary Figure S2

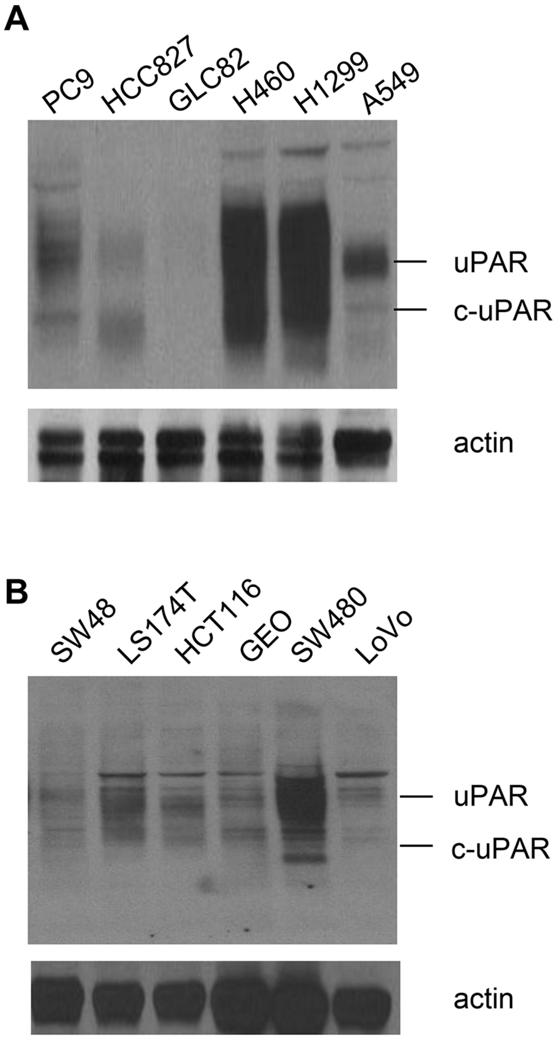

Supplementary Figure S3

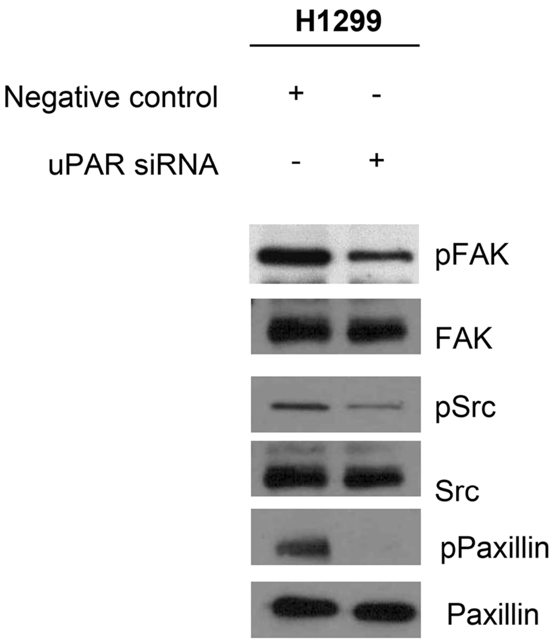

Supplementary Figure S4

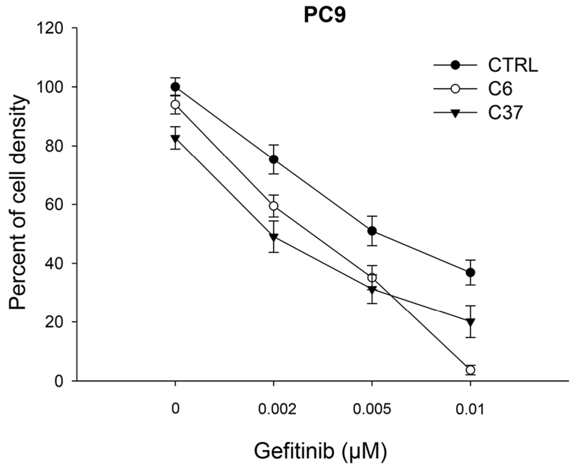

Supplementary Figure S5

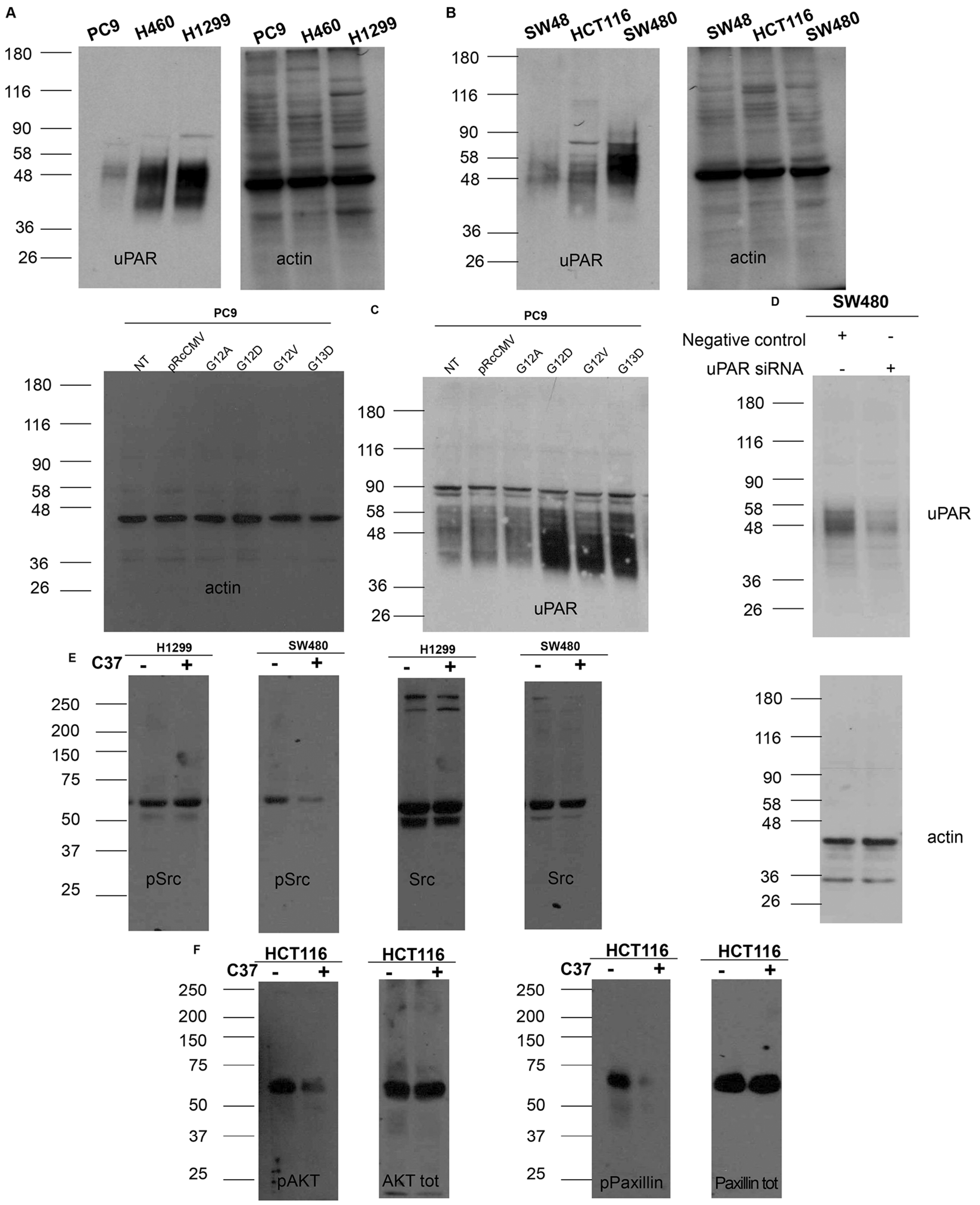

SUPPLEMENTARY FIGURE LEGENDS

Supplementary Figure S1. uPAR expression analysis in NSCLC and CRC patients. (A) Immunohistochemical images representing RAS mutated uPAR positive (Score >3) and RAS wild-type negative NSCLC (Score=0) tumors, respectively (4X and 20X magnification) (B) Immunohistochemical images representing RAS mutated uPAR positive (Score>3) and RAS wild-type negative (Score=0) CRC tumors, respectively (4X and 20X magnification).

Supplementary Figure S2. uPAR expression in NSCLC cell lines. Western blot analysis of uPAR and cleaved uPAR (c-uPAR) in NSCLC (**A**) and colorectal cancer (**B**) cell lines, both RAS wild-type and RAS mutated.

Supplementary Figure S3. Characterization of focal adhesion signals after uPAR silencing. Western blot analysis of pFAK/FAK, pSrc/Src and pPaxillin/Paxillin in response to uPAR silencing in H1299 cells.

Supplementary Figure S4. Effects of two uPAR inhibitors C6 and C37 combined with EGFR inhibitor. Percent of cell density in PC9 cells treated with increasing doses of gefitinib (0.002 to 0.01 μM) in presence of C6 or C37 (either 0.5 μM). Data represent the mean (±S.D.) of three independent experiments, each performed in triplicate, compared with control (cells treated with DMSO).

Supplementary Figure S5. Full-length scanned images of immunoblot film. Originals for cropped bands for (**A**) NSCLC cell lines and (**B**) CRC cell lines in figure 1A; (**C**) original images

for figure 2A; (**D**) originals for figure 2D; (**E**) originals for cropped band for pSrc and Src in figure 4A and figure 4B; (**F**) originals for pAKT, AKT, pPaxillin and paxillin in figure 5C. Protein ladders were used to estimate molecular weight (kilodaltons).

SUPPLEMENTARY TABLES

Supplementary Table S1. NSCLC and CRC patients features

NSCLC	uPAR -	uPAR+	pvalue	uPAR score < 3	uPAR score >=3	pvalue
Gender			0,339			0,291
Female	34 (26.2)	24 (32.4)		43 (26.7)	15 (34.9)	
Male	96 (73.8)	50 (67.6)		118 (73.3)	28 (65.1)	
T			0,062			0,669
T1	30 (27.8)	9 (12.5)		31 (22.3)	8 (19.5)	
T2	35 (32.4)	33 (45.8)		49 (35.3)	19 (46.3)	
T3	36 (33.3)	23 (31.9)		48 (34.5)	11 (26.8)	
T4	7 (6.5)	7 (9.7)		11 (7.9)	3 (7.3)	
G			0,103			0,686
G1	1 (0.9)	0 (0)		1 (0.7)	0 (0)	
G2	61 (54.5)	50 (68.5)		84 (58.7)	27 (64.3)	
G3	50 (44.6)	23 (31.5)		58 (40.6)	15 (35.7)	
N			0,342			0,864
N0	50 (54.9)	25 (43.1)		60 (50)	15 (51.7)	
N1	18 (19.8)	13 (22.4)		26 (21.7)	5 (17.2)	
N2	23 (25.3)	20 (34.5)		34 (28.3)	9 (31)	
KRAS			<0.001			<0.001
MUT	50 (39.4)	49 (67.1)		66 (42)	33 (76.7)	
WT	77 (60.6)	24 (32.9)		91 (58)	10 (23.3)	
MUT			0,243			
G12A	8 (16)	3 (6.1)		8 (12.1)	3 (9.1)	
G12C	16 (32)	22 (44.9)		24 (36.4)	14 (42.4)	
G12D	7 (14)	11 (22.4)		8 (12.1)	10 (30.3)	
G12V	10 (20)	8 (16.3)		13 (19.7)	5 (15.2)	
ALTRO	9 (18)	5 (10.2)		13 (19.7)	1 (3)	
MUT			0,496			0,554
G12A	8 (16)	3 (6.1)		8 (12.1)	3 (9.1)	
G12C	16 (32)	22 (44.9)		24 (36.4)	14 (42.4)	
G12D	7 (14)	11 (22.4)		8 (12.1)	10 (30.3)	
G12F	1 (2)	1 (2)		1 (1.5)	1 (3)	
G12L	1 (2)	0 (0)		1 (1.5)	0 (0)	
G12R	1 (2)	0 (0)		1 (1.5)	0 (0)	
G12S	1 (2)	1 (2)		2 (3)	0 (0)	
G12V	10 (20)	8 (16.3)		13 (19.7)	5 (15.2)	
G13C	2 (4)	2 (4.1)		4 (6.1)	0 (0)	
G13D	2 (4)	0 (0)		2 (3)	0 (0)	
G13P	1 (2)	0 (0)		1 (1.5)	0 (0)	
Q61H	0 (0)	1 (2)		1 (1.5)	0 (0)	

CRC	uPAR -	uPAR +	pvalue	uPAR score < 3	uPAR score >=3	pvalue
**Gender**			**0,862**			**0,487**
**Female**	**26 (43.3)**	**20 (41.7)**		**34 (44.7)**	**12 (37.5)**	
**Male**	**34 (56.7)**	**28 (58.3)**		**42 (55.3)**	**20 (62.5)**	
**T**			**0,274**			**0,638**
**T2**	**5 (8.3)**	**1 (2.1)**		**5 (6.6)**	**1 (3.2)**	
**T3**	**49 (81.7)**	**38 (80.9)**		**60 (78.9)**	**27 (87.1)**	
**T4**	**6 (10)**	**8 (17)**		**11 (14.5)**	**3 (9.7)**	
**G**			**0,062**			**0,766**
**G1**	**1 (1.7)**	**0 (0)**		**1 (3.2)**	**0 (0)**	
**G2**	**29 (48.3)**	**32 (68.1)**		**42 (55.3)**	**19 (61.3)**	
**G3**	**30 (50)**	**15 (31.9)**		**33 (43.4)**	**12 (38.7)**	
**N**			**0,881**			**0,859**
**N0**	**15 (31.9)**	**13 (12.3)**		**20 (64.5)**	**8 (7.5)**	
**N1**	**22 (37.3)**	**19 (40.4)**		**30 (40)**	**11 (35.5)**	
**N2**	**22 (37.3)**	**15 (31.9)**		**25 (33.3)**	**12 (38.7)**	
**KRAS**			**<0.001**			**0,039**
**MUT**	**18 (30)**	**33 (68.8)**		**31 (40.8)**	**20 (62.5)**	
**WT**	**42 (70)**	**15 (31.3)**		**45 (59.2)**	**12 (37.5)**	
**MUT**			**0,587**			**0,875**
**G12C**	**1 (5.6)**	**6 (18.2)**		**3 (9.7)**	**4 (20)**	
**G12D**	**7 (38.9)**	**13 (39.4)**		**12 (38.7)**	**8 (40)**	
**G12V**	**4 (22.2)**	**5 (15.2)**		**6 (19.4)**	**3 (15)**	
**G13D**	**5 (27.8)**	**5 (15.2)**		**7 (22.6)**	**3 (15)**	
**ALTRO**	**1 (5.6)**	**4 (12.1)**		**3 (9.7)**	**2 (10)**	
**MUT**			**0,521**			**0,925**
**G12A**	**1 (5.6)**	**1 (3)**		**1 (3.2)**	**1 (5)**	
**G12C**	**1 (5.6)**	**6 (18.2)**		**3 (9.7)**	**4 (20)**	
**G12D**	**7 (38.9)**	**13 (39.4)**		**12 (38.7)**	**8 (40)**	
**G12S**	**0 (0)**	**3 (9.1)**		**2 (6.5)**	**1 (5)**	
**G12V**	**4 (22.2)**	**5 (15.2)**		**6 (19.4)**	**3 (15)**	
**G13D**	**5 (27.8)**	**5 (15.2)**		**7 (22.6)**	**3 (15)**	

Supplementary Table S2. **NSCLC and CRC cell lines features**

Tumor type	Cell line	RAS status [1-2]
NSCLC	PC9	WT
	HCC827	WT
	GLC82	WT
	H460	KRAS (Q61H)
	H1299	NRAS (Q61K)
	A549	KRAS (G12S)
CRC	SW48	WT
	LS174T	KRAS (G12D)
	HCT116	KRAS (G13D)
	GEO	KRAS (G12A)
	SW480	KRAS (G12V)
	LOVO	KRAS (G13D)

1. American Type Culture Collection (ATCC, Manassas, VA, USA). **2.** Emily F. Dunn, Mari Iida, Rebecca A. Myers, Kylee A. Hintz, David A. Campbell, Eric A. Armstrong, Chunrong Li, and Deric L. Wheeler1. Dasatinib sensitizes KRAS mutant colorectal tumors to cetuximab. Oncogene. 2011 Feb 3; 30(5): 561–574.

Supplementary Table S3. Mean fluorescence intensity

Tumor type	Cell line	Non Immune IgG	Anti-uPAR Ab	Ratio
**NSCLC**	**PC9**	3,39	21,35	6,30
	**H460**	4,50	106,65	23,70
	**H1299**	2,03	28,42	14,00
**CRC**	**SW48**	5,44	16,58	3,00
	**HCT116**	3,78	13,68	3,60
	**SW480**	3,61	26,15	7,20

Supplementary Table S4. Secondary effects observed in mice treated with C37

	Diarrhea	Weight Loss ≥ 10%	Rash	Behavior disorder
**Untreated mice**	0/6	1/6	0/6	1/6
**Mice treated with C37**	0/6	2/6	0/6	2/6

Supplementary Table S5. Immunohistochemical scores for different markers on HCT116 tumor xenografts

	**Control**		**C37**	
**Vimentin**	Positive areas	Negative areas	Positive areas	Negative areas
	90% / 3+	25% / 3+	80% / 2+	5% / 2+
**E-Cadherin**	75% / 1+		90% / 2+	
**Ki67**	80%		80%	

SUPPLEMENTARY METHODS

Immunohistochemical analysis

For immunohistochemical analysis of human tumor samples, paraffin slides were de-paraffinized in xylene and rehydrated through graded alcohols. Antigen retrieval was performed with slides heated in 0.01M citrate buffer in a bath for 20 min at 97ºC. After antigen retrieval, the slides were allowed to cool. The slides were rinsed with TBS and the endogenous peroxidase was inactivated with 3% hydrogen peroxide. After protein block (BSA 5% in PBS 1x), the slides were incubated with primary antibody to uPAR (Rabbit Polyclonal anti-uPAR antibody, Novus Biologicals, CO, USA) for 30 minutes. Sections were rinsed in TBS and incubated for 20 min with Biotinylated Secondary Antibody (RE7103, Novocastra, Nussloch GmbH, Germany), a biotin-conjugated secondary antibody formulation that recognized rabbit immunoglobulins. Then the sections were rinsed in TBS and incubated for 20 min with Streptavidin-HRP (Novocastra, Newcaste, UK) and then peroxidase reactivity was visualized using a 3,3'-diaminobenzidine (DAB). Finally, the sections were counterstained with hematoxylin and mounted. Results are interpreted using a light microscope. Ten fields on each of two cores and at least >500 cells were analyzed for each sample.

For immunohistochemical analysis of mice tumor samples, the formalin fixed tumor tissue was paraffin embedded and for each sample four section were cut by microtome (4 Micron thickness) and put on adhesion microscope slides. One glass was stained with Hematoxylin & Eosin for the morphology evaluation. The immunoistochemestry was performed using the following primary antibodies: rabbit monoclonal anti-human Vimentin (clone V9); rabbit monoclonal anti-human E-Cadherin (clone EP700Y); rabbit monoclonal anti-human Ki67 (clone 30.9). No dilution was necessary because the antibody are ready to use. The signal was developed using the polymeric U-View DAB Detection kit on Benchmark XT platform (Ventana-Roche) according to a consolidated protocol. The score was made by two different pathologists: for Vimentin and E-Cadherin was

considering both the number of positive tumor cells (%) and the intensity of the signal (+); for ki67 was considered only the percentage of positive cells as a consolidated score model (1).

Cell cultures and cell lines authentication

NSCLC cells were routinely grown in RPMI-1640 medium; SW48 were grown in DMEM; LS174T, HCT116, GEO, SW480 and LoVo were grown in McCOY; all the media were supplemented with GlutaMAX, 10% fetal bovine serum (FBS), 100 U/ml penicillin and 100 μg/ml streptomycin (all from Invitrogen, Carlsbad, CA, USA).

Cell lines authentication was obtained using Short tandem repeat (STR) profiles of cell lines; STR profiles were obtained using nine highly polymorphic STR loci plus amelogenin (Cell IDTM System, Promega, Madison, WI, USA). The amplified fragments were analyzed with the ABI PRISM 3100 Genetic Analyzer. Data analysis was performed by GeneMapper® software, version 4.0. Cell lines authentication was performed by IRCCS Azienda Ospedaliera Universitaria San Martino – Istituto Nazionale per la Ricerca sul Cancro (Genova, Italy). The cells were last tested between April and August 2015.

RAS gene mutagenesis

The wild-type and mutant RAS expression vectors were generated by site-directed mutagenesis with the QuikChange™ II XL kit (Agilent, CA, USA). The starting template was the $KRAS_{G12V}$ mutant cDNA in the pRcCMV vector, from which the other mutant clones for codon 12 were individually derived. Oligonucleotide primers were obtained from the CEINGE oligonucleotide synthesis facility (Naples, Italy). Their sequences are indicated: $KRAS_{V12D}$ Forward5'-GGTAGTT GGAGCTGCTGGCGTAGGCAAGA; $KRAS_{V12D}$ Reverse 5'CTCTTGCCTACGCCATCAGCTC CAACTACCA; $KRAS_{V12A}$ Forward 5'GGTAGTTGGAGCTGCTGGCGTAGGCAAGA; $KRAS_{V12A}$ Reverse 5'TCTTGCCTACGCCAGCAGCTCCAACTACC.

The RAS mutant G13D was derived from the wild-type expression vector, described above, with the following oligonucleotide primers: $KRAS_{G13D}$ Forward 5'-TAGTTGGAGCTGGTGACGTAGG CAAGAGTGC; RAS_{G13D} Reverse 5'-GCACTCTTGCCTACGTCACCAGCTCCAACTA.

RAS mutant transfection

2×10^5 RAS wild-type PC9 and SW48 cells were cultured respectively in RPMI and DMEM medium supplemented with 10% FBS, 100 U/ml penicillin and 100 µg/ml streptomycin (all from Invitrogen) and grown at 37 °C in a humidified atmosphere containing 5% CO_2. For transfection, 2µg of either pRcCMV-KRAS G12A, pRcCMV-KRAS G12D, pRcCMV-KRAS G12V, pRcCMV-KRAS G13A or pRcCMV-empty vector were added together with Lipofectamine 2000 Reagent (Invitrogen), following the manufacturer's instructions. SW48 stable cell lines were established by selection of positive transfected clones that grew in media containing 1 mg/ml of G418 for two weeks. Isolated clones were picked and individually amplified for western blot analysis.

Immunofluorescence

For immunofluorescence assay on cancer cells, H1299 and SW480 cells (3x104/well) were plated in 24-well plates, previously covered with a glass cover slide (BD Biosciences, San Jose, CA, USA). Then, cells were treated with C37 (2.5 µM). After 24 hours of treatment, cells were fixed with freshly prepared 4% paraformaldehyde in PBS, permeabilized with 0.2% Triton X-100 for 5 min, and then rinsed three times with 1% bovine serum albumin in PBS. Cells were then incubated with the primary antibody p-Paxillin (1:50) for 1 hour at room temperature (Cell Signaling Technologies), and then with a fluorescent Cy2-AffiniPure Donkey Anti-Rabbit IgG secondary antibody (LiStarFish, Milan, Italy) for 1 hour at room temperature. Phalloidin-TRITC (25 mg/ml) was obtained from Sigma-Aldrich (St Louis, MO, USA) and was used to visualize the actin cytoskeleton organization. Nuclei are stained in blue with Hoechst 33342 (1:25000) (Life Technologies) for 10 min at room temperature. Slides were mounted with 50% glycerol in PBS and

imaged with a Zeiss LSM 510 meta confocal microscope (Zeiss, Oberkochen, Germany) equipped with an oil immersion plan apochromat _63 objective 1.4 NA.

Sample preparation and Chromatographic conditions

All chemicals and reagents used for pharmacokinetics study were purchased from Delchimica (Naples, Italy). C37 serum concentrations were determined by Liquid Chromatography (LC) validated method. Briefly, rat serum samples (200 µL) were added of a volume of 25% w/v perchloric acid (200 µL) to release the analytes from plasma proteins and 150 µL of a mixture of acetonitrile/phosphate buffer 0.025 M pH 3.0 55:45 (v/v). After 10s vortexing, the mixture was centrifuged at 5000 x g for 15 minutes; the clear supernatant was collected in a new tube and analyzed by LC. The LC system was a chromatograph LC-10 AD VP model (Shimadzu Corp., Kyoto, Japan), equipped with a 7725 Rheodyne injection valve fitted with a 20 µL loop. The stainless steel column was a reversed-phase Supelco Ascentis C18 (250× 4.6 mm, 5.0 µm i.d.) LC column, with a Supelguard Ascentis C18 guard column (both from Supelco, Bellefonte, PA, USA). C37 was eluted in isocratic mode using a mixture acetonitrile/phosphate buffer 0.025 M pH 3.0 55:45 (v/v) as mobile phase (flow rate 0.8 mL/min). The detection was performed by UV light on a spectrophotmeter model SPD 10 AV UV Vis (Shimadzu Corp.), set at λ of 250 nm. The chromatographic data were processed by Cromatoplus 2008 software (Shimadzu Corp.). Under the above conditions the retention time (tr) was 7.25 ± 0.30 min. The system was calibrated by using standard solutions prepared by diluting C37 in mobile phase to the final concentrations ranging from 2.5 to 12.5 µg/mL. Under our experimental conditions, the limits of quantification (LOQ: signal/noise ratio = 10) and of detection (LOD: signal/noise ratio = 3) were 0.582 µg/mL and 0.175 µg/mL. The accuracy of the method was evaluated via recovery of C37 at the concentration level of 12.5 µg/mL. The recovery was found out to be 79.3 %. Three replicates of each sample were analyzed.

Cell Adhesion assay

Adhesion assays were conducted on 96-well flat bottomed plates for cell (Nunc, Roskild, Denmark) coated with 0,25 μg (NSCLC cell lines) or 1 μg (CRC cell lines) of VN (BD Biosciences, Franklin Lakes, NJ, USA) or with 100 μl of heat-denatured 1% bovine serum albumin (BSA) in PBS, as a negative control, and incubated overnight at 4 °C. The plates were then blocked for 1 hour at room temperature with 1% heat-denatured BSA in PBS. Cells were detached with trypsin, resuspended in their culture medium and incubated for 1 hour at 37 °C, 5% CO2, to allow receptor recovery. Cells were then washed with serum-free medium, counted, plated into the wells at a density of 5x104 cell per well (NSCLC cell lines) and 105cells per well (CRC cell lines), and incubated for 1 hour at 37 °C. Attached cells were fixed with 3% paraformaldehyde in PBS for 10 minutes and then incubated with 2% methanol for 10 minutes. Cells were finally stained for 10 minutes with 0.5% crystal violet in 20% methanol. Stain was eluted by 0.1 mol/L sodium citrate in 50% ethanol, pH 4.2, and the absorbance at 540 nm was measured by a spectrophotometer.

Migration assay

Cell migration assays were performed using Transwell polycarbonate filters (surface area, 0.3 cm^2; pore size, 0.8 μm ; BD Biosciences). The bottom of wells in a 24-multiwell plate were coated with VN(10ug/ml) or with heat-denatured 1% BSAin PBS, as a negative control, and incubated overnight at 4°C; then NSCLC and CRC cells ($3x10^4$ cell/well) were seeded on filters in serum-free media. After 48 hours the upper cell layer was removed with a cotton swab and cells on the underside of the transwell were fixed and stained with crystal violet. Absorbance at 540nm was measured by a spectrophotometer.

Statistical analysis

The Student's t test was used to evaluate the statistical significance of the *in vitro* results. The linear regression test was used to evaluate the statistical significance of the cell density assays with

combination of uPAR inhibitors and EGFR inhibitor (Graph-Pad version 5). The Pearson χ^2 test was used to determine whether a relationship exists between uPAR expression and RAS mutations as measured by immunohistochemical analysis on NSCLC and CRC tumor samples. Sensitivity and Specificity of uPAR expression were computed using standard formulae while the corresponding 95% Confidence Interval (95% C.I) was based on a bootstrap approach. All reported P values were two-sided. Statistical Analysis was carried out using Statistical Package for Social Science v. 20 software (SPSS Inc., Chicago, IL) and R statistical computing software (R Foundation for Statistical Computing, Vienna, Austria).

1. Inti Zlobec et al. Selecting immunohistochemical cut−off scores for novel biomarkers of progression and survival in colorectal cancer. *J Clin Pathol.* **60**(10): 1112–1116 (2007).
